# Supplementary material for: A conserved N-terminal motif of CUL3 contributes to assembly and E3 ligase activity of CRL3KLHL22
Source: Nat Commun. 2024 May 6;15:3789. doi: 10.1038/s41467-024-48045-2 (PMC11074293; doi:10.1038/s41467-024-48045-2)
Supplement: Supplementary file 1 — Supplementary Information [file 41467_2024_48045_MOESM1_ESM.pdf]

Supplementary Information File

A conserved N-terminal motif of CUL3 contributes to assembly and E3 ligase activity of CRL3^{KLHL22}

Weize Wang, Ling Liang*, Zonglin Dai, Peng Zuo, Shang Yu, Yishuo Lu, Dian Ding, Hongyi Chen, Hui Shan, Yan Jin, Youdong Mao, Yuxin Yin*

*Correspondence: yinyuxin@hsc.pku.edu.cn (Y.Y.)., liangling@bjmu.edu.cn

The PDF file includes:
Supplementary Figures 1-8
Supplementary table 1 | Cryo-EM data collection, refinement and validation statistics
Supplementary table 2 | Primers used for plasmids construction

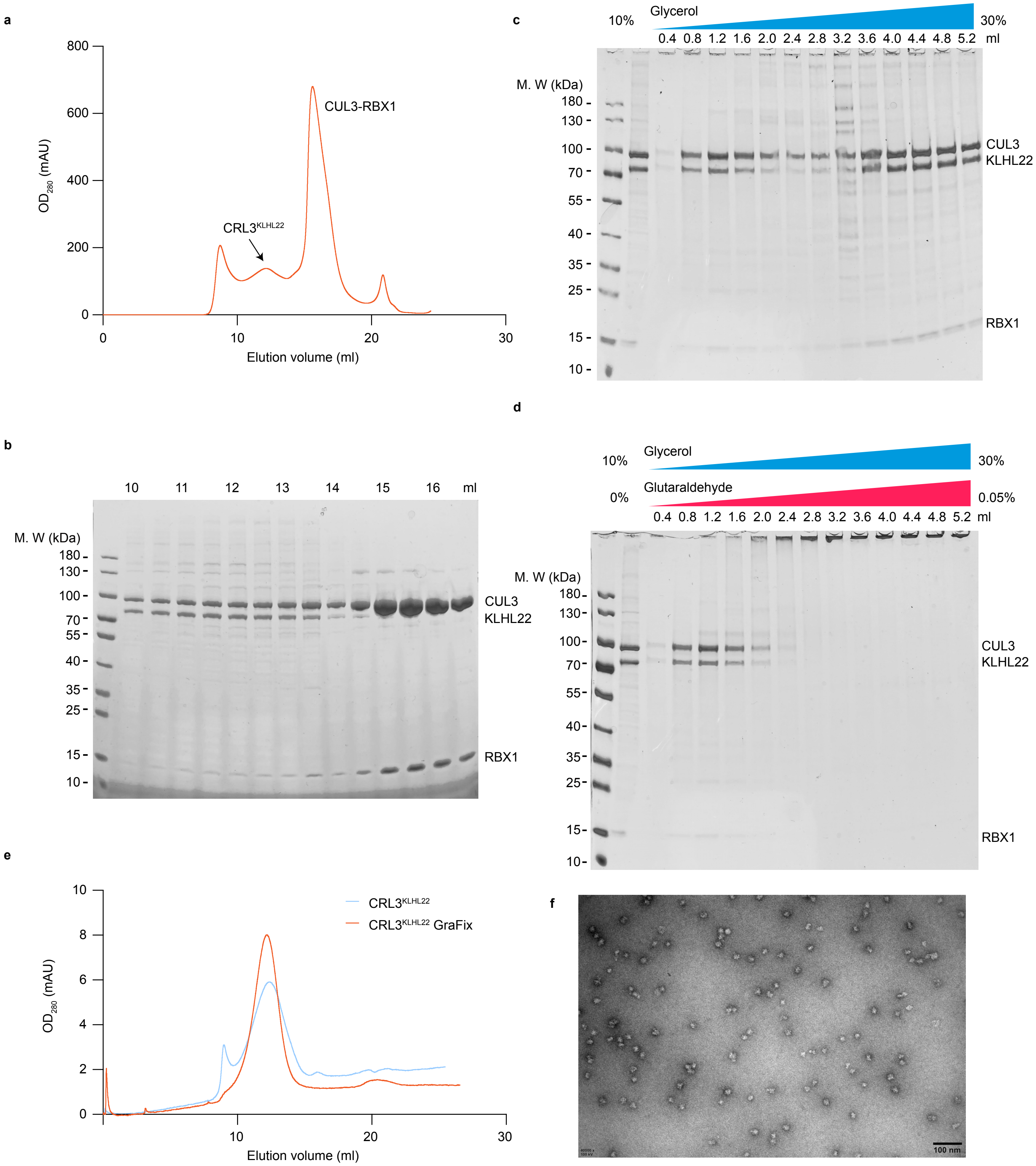

Supplementary Fig. 1 | See next page for caption

Supplementary Fig. 1 | CRL3^{KLHL22} complex preparation for cryo-EM reconstruction

a, b Co-expression and purification of full-length KLHL22, CUL3, and RBX1 from SF9 cells. The chromatogram shows the separation of the CRL3^{KLHL22} complex from the affinity purified sample. The CRL3^{KLHL22} and CUL3-RBX1 complexes were eluted at 12.5 ml and 16 ml, respectively, from the Superose6 column (**a**). SDS-PAGE gel shows the purity and expected molecular sizes of KLHL22, CUL3, and RBX1 (**b**). Source data are provided as a Source Data file.

c, d Optimization of CRL3^{KLHL22} complex using GraFix. SDS-PAGE gels show the uncross-linked (**c**) and glutaraldehyde cross-linked (**d**) CRL3^{KLHL22} complex.

e SEC analysis of of CRL3^{KLHL22} complex homogeneity before (blue line) and after (red line) GraFix. Source data are provided as a Source Data file.

f Negative stain EM of CRL3^{KLHL22} complex. Scale bar: 100 nm.

For **a**, **b**, **c**, **d**, and **e**, source data are provided as a Source Data file.

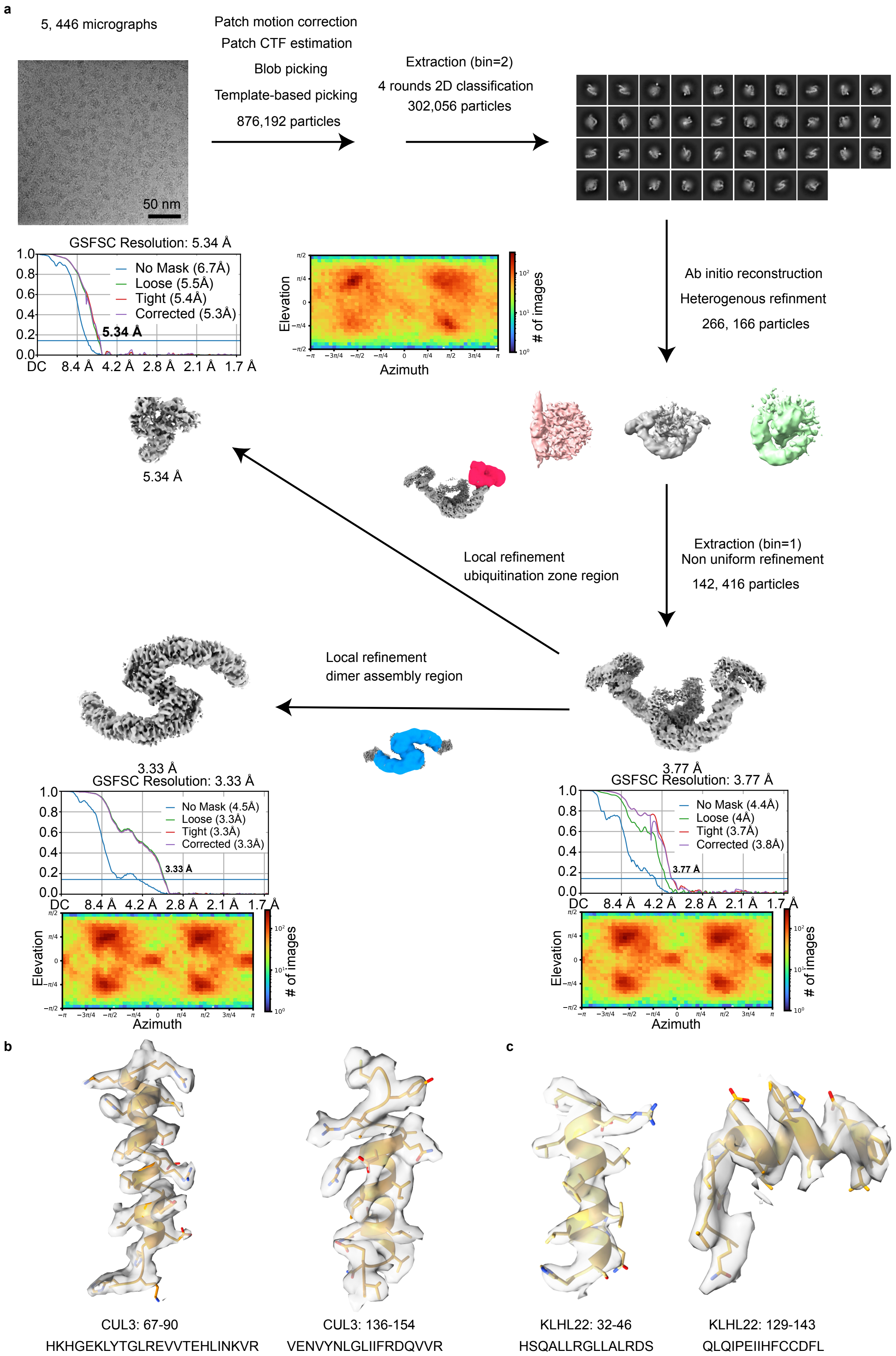

Supplementary Fig. 2 | See next page for caption

Supplementary Fig. 2 | Cryo-EM processing workflow of CRL3^{KLHL22}

a Flow chart outlining the 3D reconstruction of CRL3^{KLHL22} using cryo-EM data, related FSC curve and the particles angular distribution of the final density map. Reconstruction details are described in Methods.

b, c Representative region of EM density map of CUL3 (**b**) and KLHL22 (**c**) with corresponding stick models superimposed.

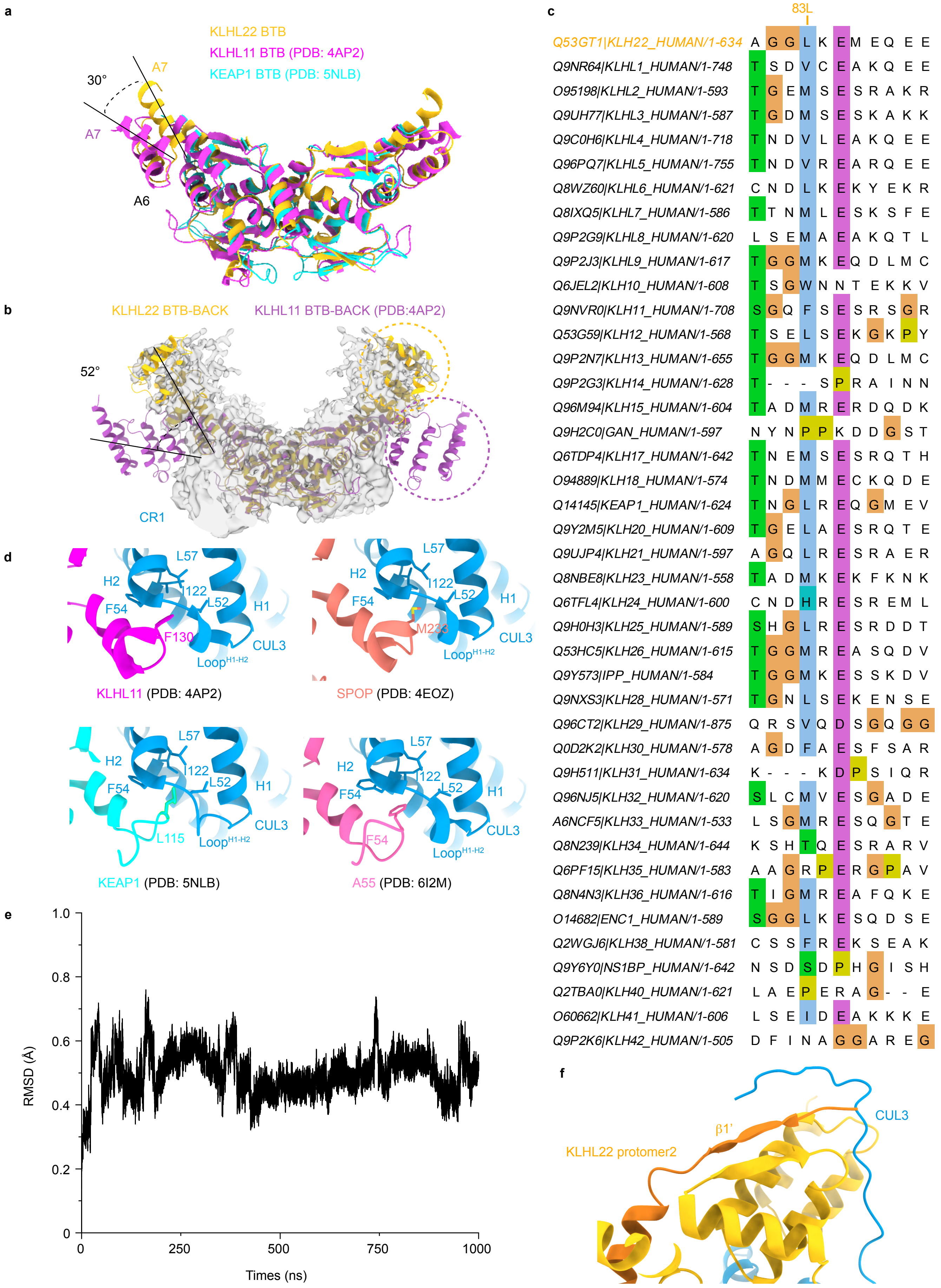

Supplementary Fig. 3 | See next page for caption

Supplementary Fig. 3 | Both the hydrophobic core and CUL3 NA motif participate in dimeric CRL3^{KLHL22} assembly

a Alignment of KLHL22 BTB with KLHL11 (PDB: 4AP2) and KEAP1 (PDB: 5NLB). The BTB core fold and α-helix A6 of KLHL22 aligned well with those of KLHL11. The α-helix A7 of KLHL22 displays a 30° rotation relative to the orientation of α-helix A7 from KLHL11.

b The BTB-BACK domain of KLHL22 is shaped like a fluttering bird.

c Evolutionary conservation analysis showing that the residues corresponding to KLHL22-Leu83 is almost always a hydrophobic residue within other KLHL family members. The human KLHL (1-42) sequences were aligned using Jalview[62] and Clustal software[63].

d Hydrophobic core formed by the BTB domain (KLHL11, SPOP, KEAP1, A55) and the CR1 domain of CUL3. Interfacial residues are shown.

e, f Analysis of the binding model between the CUL3 NA motif (aa 2-13) and KLHL22 by molecular dynamics (MD) simulation. Root mean square deviation (RMSD) of CUL3$^{1\text{-}155}$-KLHL22$^{22\text{-}178}$ with respect to the initial structure during a 1000 ns simulation (**e**). The average structures from 800 ns to 1000 ns of MD trajectory are shown (**f**). Source data are provided as a Source Data file.

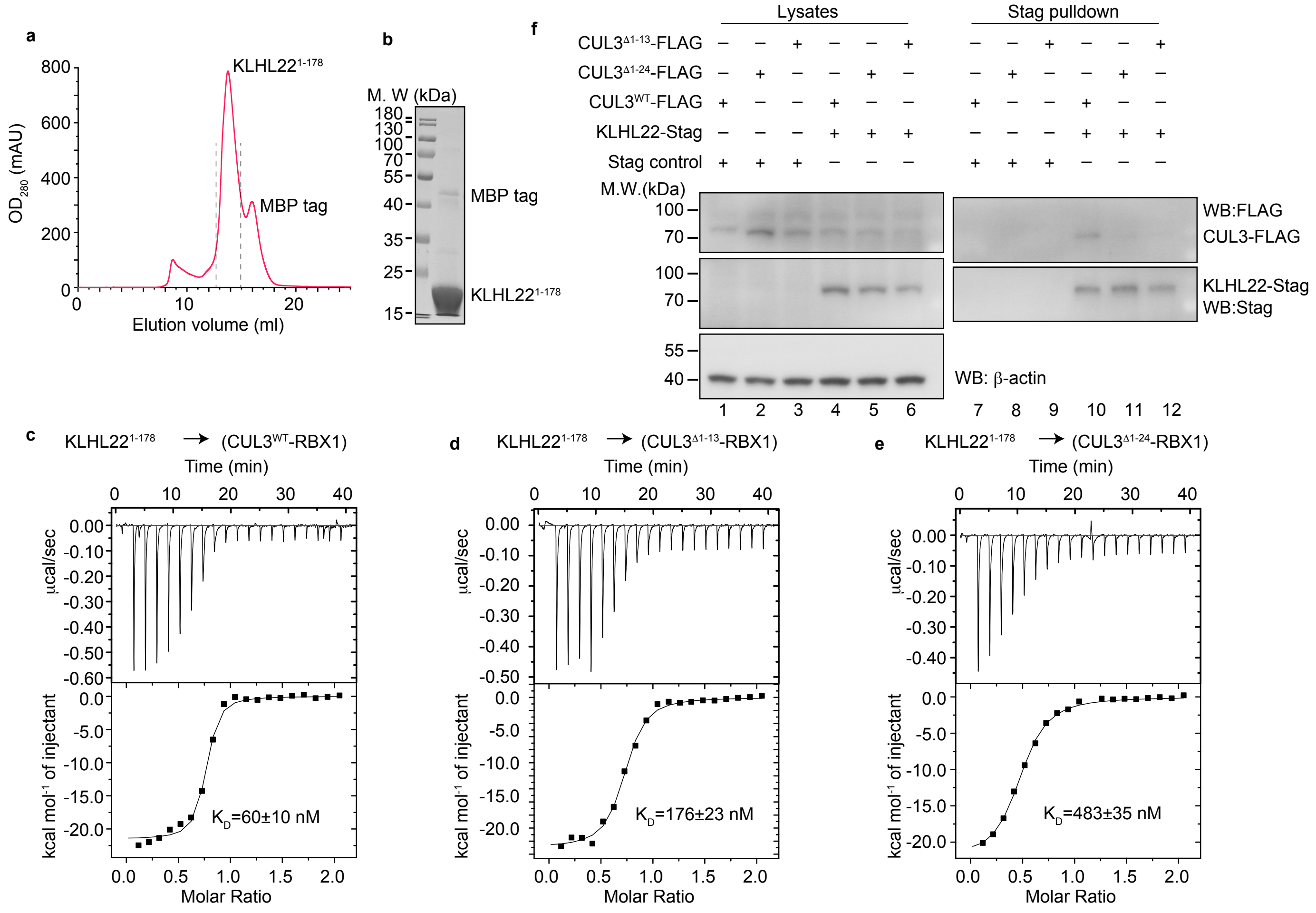

Supplementary Fig. 4 | CUL3 NA motif participates in the CRL3^{KLHL22} assembly

a, b Purification of KLHL22$^{1\text{-}178}$. Chromatogram showing the separation of KLHL22$^{1\text{-}178}$ from the affinity-purified sample (**a**). SDS-PAGE gel of purified KLHL22$^{1\text{-}178}$ (**b**).

c-e ITC measurement of binding affinity between KLHL22$^{1\text{-}178}$ and CUL3WT (**c**), CUL3$^{\Delta1\text{-}13}$ (**d**), and CUL3$^{\Delta1\text{-}24}$ (**e**), respectively. KLHL22$^{1\text{-}178}$ was titrated into the cell containing CUL3-RBX1 (wild-type or NA motif truncation mutants). The binding constants (KD ± SD) are indicated.

f Deletion of the CUL3 NA motif impairs interaction between CUL3 and KLHL22 in intact cells. S-tag control or KLHL22^{WT}-(S-tag) was co-expressed with CUL3^{WT}-FLAG, CUL3$^{\Delta1\text{-}13}$-FLAG, and CUL3$^{\Delta1\text{-}24}$-FLAG in HEK 293T cells, and S-tag pulldown assays were performed.

For **a**, **b**, **c**, **d**, **e**, and **f**, source data are provided as a Source Data file.

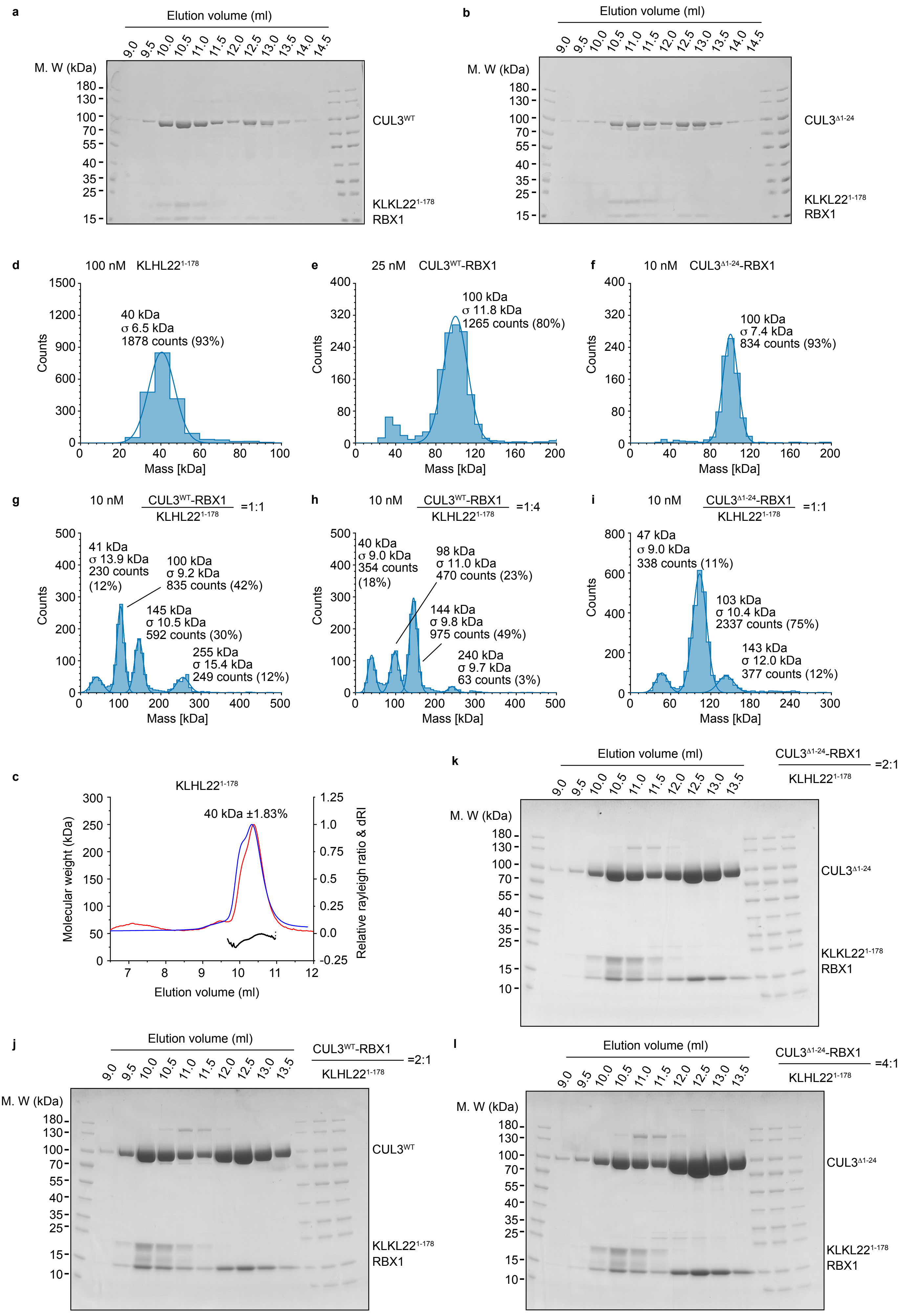

Supplementary Fig. 5 | See next page for caption

Supplementary Fig. 5 | CUL3 NA motif participates in the CRL3^{KLHL22} assembly

a-b Representative SDS-PAGE gel of CUL3^{WT}-RBX1-KLHL22$^{1\text{-}178}$ (**a**), and CUL3$^{\Delta 1\text{-}24}$-RBX1-KLHL22$^{1\text{-}178}$ complex (**b**) analyzed in SEC fraction in Fig. 4f. The results shown are representative of two biological replicates. Source data are provided as a Source Data file.

c SEC-MALS analysis of the dimeric KLHL22$^{1\text{-}178}$. The chromatograms show the relative Rayleigh ratios (right Y-axes, red line), relative dRI ratios (right Y-axes, blue line), and calculated molecular weights (left Y-axes, black line).

d-i Mass photometry analysis of the effect of CUL3 NA motif deletion on the assembly of the CUL3-RBX1-KLHL22$^{1\text{-}178}$. Shown analyses are representative of at least three replicate experiments: KLHL22$^{1\text{-}178}$(**d**, 100 nM), CUL3^{WT}-RBX1(**e**, 25 nM), CUL3$^{\Delta 1\text{-}24}$-RBX1(**f**, 10 nM), CUL3^{WT}-RBX1-KLHL22$^{1\text{-}178}$ (**g,** 10 nM CUL3^{WT}-RBX1 incubate with 10 nM KLHL22$^{1\text{-}178}$), CUL3^{WT}-RBX1-KLHL22$^{1\text{-}178}$ (**h**, 10 nM CUL3^{WT}-RBX1 incubate with 40 nM KLHL22$^{1\text{-}178}$) and CUL3$^{\Delta 1\text{-}24}$-RBX1-KLHL22$^{1\text{-}178}$ (**i**, 10 nM CUL3$^{\Delta 1\text{-}24}$-RBX1 incubate with 10 nM KLHL22$^{1\text{-}178}$). The blue line represents Gaussian fits to the peak. Above each peak, the peak mass of the Gaussian fit, the count of particles and percentage of particles in the peak are indicated.

j-l SDS-PAGE gel of CUL3^{WT}-RBX1-KLHL22$^{1\text{-}178}$ (**j**, 2:1), CUL3$^{\Delta 1\text{-}24}$-RBX1-KLHL22$^{1\text{-}178}$ complex (**k**, 2:1) and CUL3$^{\Delta 1\text{-}24}$-RBX1-KLHL22$^{1\text{-}178}$ complex (**l**, 4:1) analyzed in SEC fraction in Fig. 4j. Source data are provided as a Source Data file.

For **a**, **b**, **c**, **d**, **e**, **f**, **g**, **h**, **i**, **j**, **k** and **l**, source data are provided as a Source Data file.

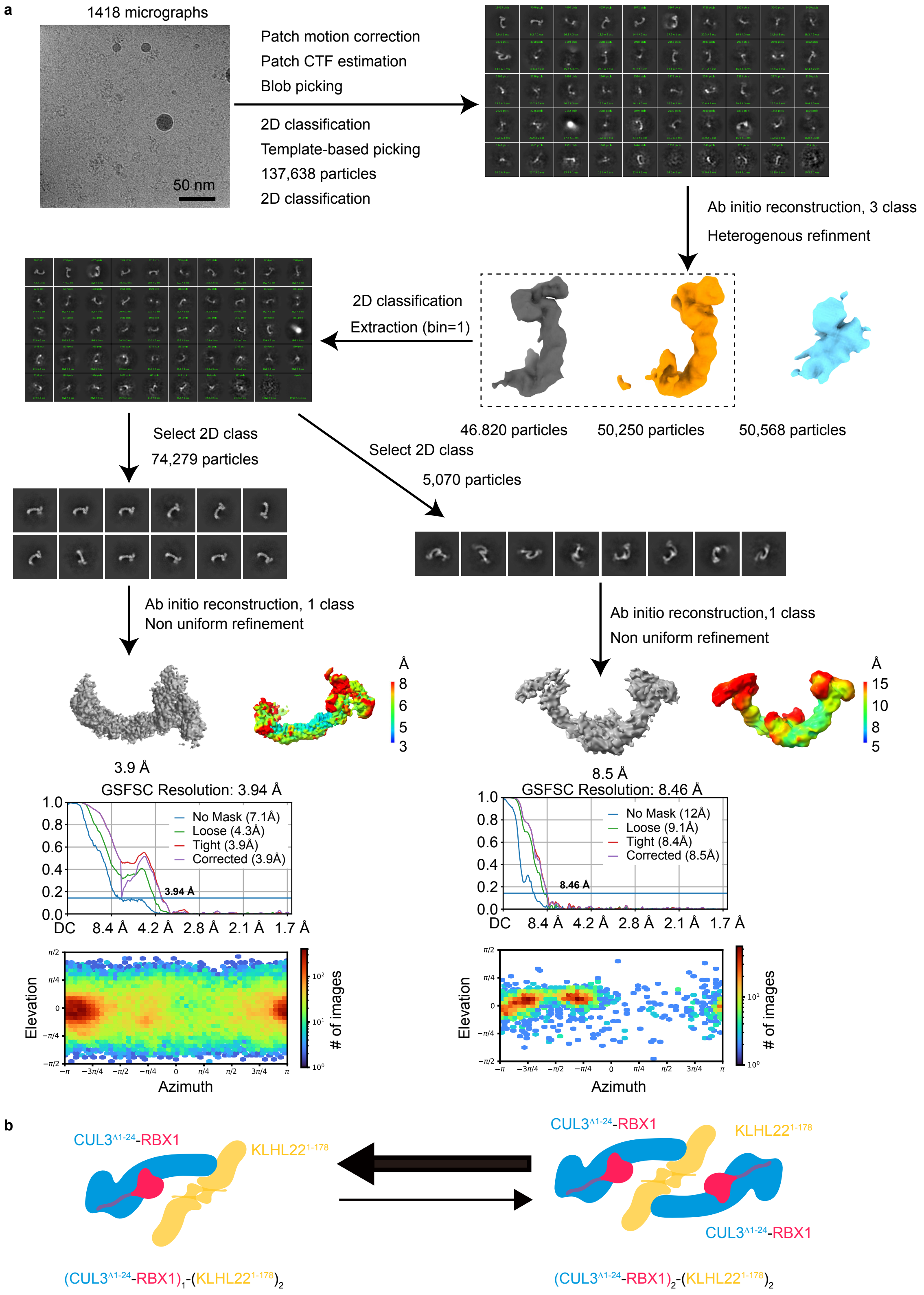
a
1418 micrographs
50 nm
Patch motion correction
Patch CTF estimation
Blob picking
2D classification
Template-based picking
137,638 particles
2D classification
Ab initio reconstruction, 3 class
Heterogenous refinment
2D classification
Extraction (bin=1)
46.820 particles
50,250 particles
50,568 particles
Select 2D class
74,279 particles
Select 2D class
5,070 particles
Ab initio reconstruction, 1 class
Non uniform refinement
Ab initio reconstruction,1 class
Non uniform refinement
3.9 Å
8.5 Å
GSFSC Resolution: 3.94 Å
No Mask (7.1Å)
Loose (4.3Å)
Tight (3.9Å)
Corrected (3.9Å)
GSFSC Resolution: 8.46 Å
No Mask (12Å)
Loose (9.1Å)
Tight (8.4Å)
Corrected (8.5Å)
DC 8.4 Å 4.2 Å 2.8 Å 2.1 Å 1.7 Å
Elevation
Azimuth
# of images
b
CUL3Δ1-24-RBX1
KLHL221-178
(CUL3Δ1-24-RBX1)1-(KLHL221-178)2
(CUL3Δ1-24-RBX1)2-(KLHL221-178)2

Supplementary Fig. 6 | Cryo-EM processing workflow of the CUL3$^{\Delta 1\text{-}24}$-RBX1-KLHL22$^{1\text{-}178}$ complex

a Flow chart outlining procedures for the 3D reconstruction of the CUL3$^{\Delta 1\text{-}24}$-RBX1-KLHL22$^{1\text{-}178}$ complex and related FSC curve of the final density map.

b A cartoon model showing the dynamic equilibrium between the (CUL3$^{\Delta 1\text{-}24}$-RBX1)$_1$-(KLHL22$^{1\text{-}178}$)$_2$ state and the (CUL3$^{\Delta 1\text{-}24}$-RBX1)$_2$-(KLHL22$^{1\text{-}178}$)$_2$ state. The equilibrium favors formation of (CUL3$^{\Delta 1\text{-}24}$-RBX1)$_1$-(KLHL22$^{1\text{-}178}$)$_2$.

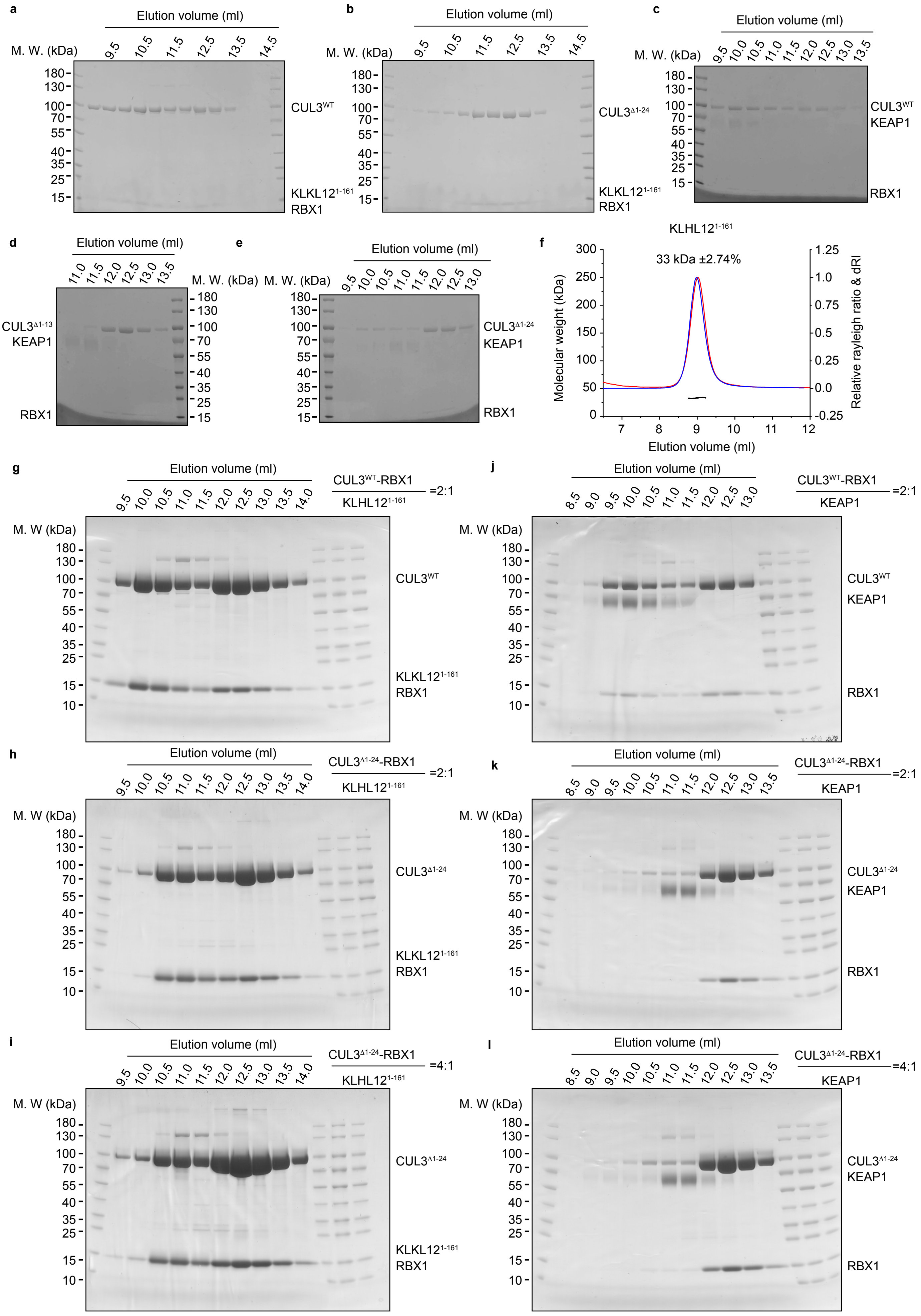

Supplementary Fig. 7 | See next page for caption

Supplementary Fig. 7 | Conserved CUL3 NA motif participates in the assembly of CRL3^{KLHL12} and CRL3^{KEAP1}

a, b Representative SDS-PAGE gels of CUL3^{WT}-RBX1-KLHL12$^{1\text{-}161}$ (**a**), and CUL3$^{\Delta1\text{-}24}$-RBX1-KL-HL12$^{1\text{-}161}$ complex (**b**) analyzed in SEC fractions shown in main Fig. 5b. The results shown are represen-tative of two biological replicates. Source data are provided as a Source Data file.

c-e Representative SDS-PAGE gels of CUL3^{WT}-RBX1-KEAP1 (**c**), CUL3$^{\Delta1\text{-}13}$-RBX1-KEAP1 (**d**), and CUL3$^{\Delta1\text{-}24}$-RBX1-KEAP1 (**e**) complexes analyzed in SEC fractions shown in main Fig. 5c. The results shown are representative of two biological replicates. Source data are provided as a Source Data file.

f SEC-MALS analysis of the dimeric KLHL12$^{1\text{-}161}$. The chromatograms show the relative Rayleigh ratios (right Y-axes, red line), relative dRI ratios (right Y-axes, blue line), and calculated molecular weights (left Y-axes, black line). Source data are provided as a Source Data file.

g-i SDS-PAGE gel of CUL3^{WT}-RBX1-KLHL12$^{1\text{-}161}$ (**g**, 2:1), CUL3$^{\Delta1\text{-}24}$-RBX1-KLHL12$^{1\text{-}161}$ complex (**h**, 2:1) and CUL3$^{\Delta1\text{-}24}$-RBX1-KLHL12$^{1\text{-}161}$ complex (**i**, 4:1) analyzed in SEC fraction in main Fig. 5f.

j-l SDS-PAGE gel of CUL3^{WT}-RBX1-KEAP1 (**j**, 2:1), CUL3$^{\Delta1\text{-}24}$-RBX1-KEAP1 complex (**k**, 2:1) and CUL3$^{\Delta1\text{-}24}$-RBX1-KEAP1 complex (**l**, 4:1) analyzed in SEC fraction in main Fig. 5g. Source data are provided as a Source Data file.

For **a**, **b**, **c**, **d**, **e**, **f**, **g**, **h**, **i**, **j**, **k**, and **l**, source data are provided as a Source Data file.

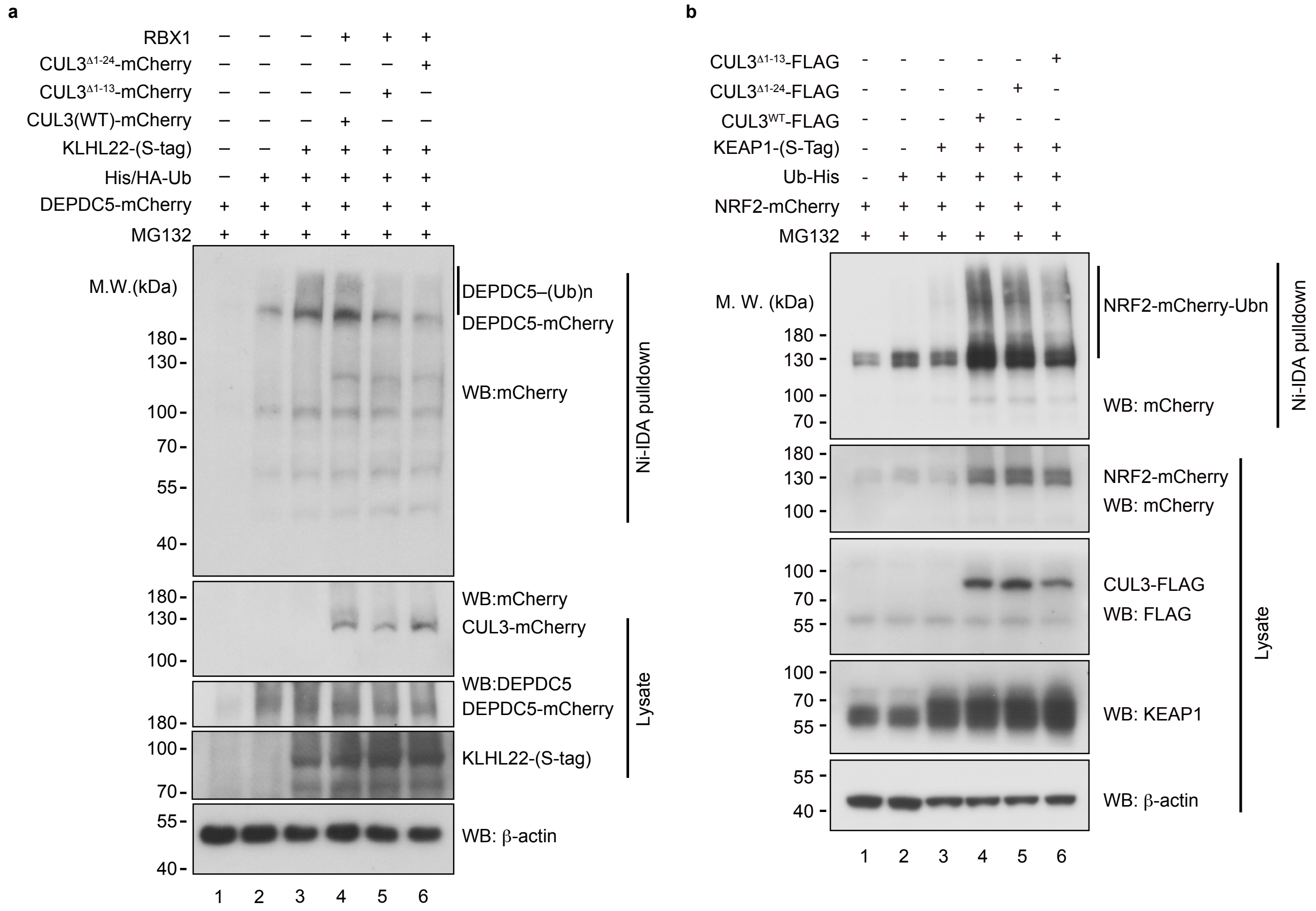

Supplementary Fig. 8 | CUL3 NA motif deletion impairs E3 ligase activity of CRL3^{KLHL22} and CRL3^{KEAP1}

a, **b** E3 activity analysis using an in vivo ubiquitination assay. The indicated plasmids were co-transfected with His-tagged ubiquitin in HEK 293T cells, and then the cells were treated with MG132 (20 μM) for 6 hr before harvesting. The cells were subjected to pulldown under denaturing conditions using Ni^{2+}-IDA agarose. Polyubiquitinated DEPDC5 (**f**) and NRF2 (**g**) (shown as a smear of high-molecular-weight polypeptides) was detected by Western Blotting with anti-mCherry antibody. The levels of CUL3, DEPDC5, NRF2, KLHL22, KEAP1 and β-actin in cell lysates are shown. Source data are provided as a Source Data file. Source data are provided as a Source Data file.

Supplementary table 1 |
Cryo-EM data collection, refinement and validation statistics

	CUL3-RBX1-KLHL22 complex (EMDB-36961) (PDB 8K8T)	CUL3-RBX1-KLHL22 complex without CUL3 NA motif (EMDB-36987) (PDB 8K9I)
**Data collection and processing**		
Magnification	81,000	81,000
Voltage (kV)	300	300
Electron exposure (e–/Å^2)	50	50
Defocus range (μm)	-1.5 to -2	-1.5 to -2
Pixel size (Å)	0.821	0.821
Symmetry imposed	C2	C1
Initial particle images (no.)	302,056	137,638
Final particle images (no.)	142,416	74,279
Map resolution (Å)	3.8	4.0
FSC threshold	0.143	0.143
Map resolution range (Å)	3.12-60.98	3.39-61.03
**Refinement**		
Initial model used (PDB code)	8K8T	8K9I
Model resolution (Å)	3.82	4.0
FSC threshold	0.143	0.143
Map sharpening *B* factor (Å^2)	-114.7	-110.6
Model composition		
Non-hydrogen atoms	9926	7658
Protein residues	1210	939
Ligands	0	0
B factors (Å^2)		
Protein	98.32	97.49
Ligand	0	0
R.m.s. deviations		
Bond lengths (Å)	0.014	0.04
Bond angles (°)	0.969	0.702
Validation		
MolProbity score	2.34	1.95
Clashscore	15.42	18.43
Poor rotamers (%)	2.79	0.24
Ramachandran plot		
Favored (%)	95.56	96.94
Allowed (%)	4.44	3.06
Disallowed (%)	0	0

Supplementary table 2 | Primers used for plasmids construction

Primer name	Sequence
CUL3-F	CCCACCATCGGGCGCGGATCCGCCACCATGTCGAATCTGAGCAAAGGCACG
CUL3-R	GTGATGGTGATGATGAAGCTTTGCTACATATGTGTATACTTTGCGATCCTCAG
CUL3-Del1-13-F	CCACCATCGGGCGCGGATCCGCCACCatgACCAAGATGCGGATCCGGGCCTTTCC
CUL3-Del1-24-F	CCCACCATCGGGCGCGGATCCGCCACCATGGATGAAAAATATGTAAACAGCATTTG
RBX1-F	TGATCACCCGGGATCTCGAGatggcggcagcgatggatg
RBX1-R	ATCAGCTGCTAGCACCATGGctagtgcccatacttttgg
HIS-KLHL22-F	TGTATTTTCAGGGCGCCATGATGGCAGAGGAGCAGGAGTTC
HIS-KLHL22-R	CTTGGTACCGCATGCCTCGAGCTAGTCCTCACTGGAGTTGTCAAACTCC
HIS-MBP-KLHL22-F	CCGAAAACCTGTATTTTCAGGGCcatatgATGGCAGAGGAGCAGGAGTTC
HIS-MBP-KLHL22-1-178-R	GTGGTGGTGGTGGTGCTCGAGTCAGTTTTTGAGGATATAGGTGTCCAGTTGCTC
HIS-MBP-KLHL12-F	TGTATTTTCAGGGCGAATTCATGGGAGGCATTATGGCCCC
HIS-MBP-KLHL12-1-161-R	GTGGTGGTGGTGGTGCTCGAGctaATGCTTCTGGCTAAAAACCTCAGCTG
HIS-KEAP1-F	TGTATTTTCAGGGCGCCATGATGCAGCCAGATCCCAGGCCTAG
HIS-KEAP1-R	CTTGGTACCGCATGCCTCGAGTCAACAGGTACAGTTCTGCTGGTCAATC
NRF2-F	TTACAGCTCTTAAGGGAATTCGCCACCATGATGGACTTGGAGCTGCCGCCGCCGGGAC
NRF2-R	AAAATACAGGTTTTCTCTAGaGTTTTTCTTAACATCTGGCTTCTTACTTTTGGGAAC
MBP-NRF2-1-100-F	AACCTGTATTTTCAGGGCGccatgATGATGGACTTGGAGCTGCCGCC
MBP-NRF2-1-100-R	GTGGTGGTGGTGGTGCTCGAGctaGGCAGATCCACTGGTTTCTGACTGGATG
DEPDC5-F	AATTCAAAGGCCTACGTCGACgccaccATGAGAACAACAAAGGTCTACAAACTCGTC
DEPDC5-R	CCCTGAAAATACAGGTTTTCTCTAGACGGGGCACTGGCATGCATCTTCTCCAGG
NEDD8-F	GCAAATGGGTCGCGGATCCATGCTAATTAAAGTGAAGACGC
NEDD8-R	CTCGAGTGCGGCCGCTCACTGCCTAAGACCACC
UBA1-F	GCAAATGGGTCGCGGATCCATGTCCAGCTCGCCGCTG
UBA1-R	CTCGAGTGCGGCCGCTCAGCGGATGGTGTATCGGAC
UBE2D1-F	TAAGAAGGAGATATACCATGATGGCGCTGAAGAGGATTCAGAAAG
UBE2D1-R	GTGGTGGTGGTGGTGCTCGAGCATTGCATATTTCTGAGTCCATTCTCTTGC

UBE1-F	GCAAATGGGTCGCGGATCCATGTCCAGCTCGCCGCTG
UBE1-R	CTCGAGTGCGGCCGCTCAGCGGATGGTGTATCGGAC
NAE1-F	GGAGATATACATATGgcgcagctgggaaagctgctc
NAE1-R	CTTTACCAGACTCGAGctacaactggaaagttgctgaagtttgtgacatg
UBA3-F	GTATTTTCAGGGAGAATTCATGGCGGATGGCGAGGAGCC
UBA3-R	GTGGTGGTGCTCGAGTTAAGAAGTAAAATGAAGTTTGAATAGTACAGTCTGTGGGG
UBE2M-F	GCAAATGGGTCGCGGATCCATGATCAAGCTGTTCTCG
UBE2M-R	CTCGAGTGCGGCCGCCTATTTCAGGCAGCGC
