# Supplementary material for: A conserved N-terminal motif of CUL3 contributes to assembly and E3 ligase activity of CRL3KLHL22
Source: Nat Commun. 2024 May 6;15:3789. doi: 10.1038/s41467-024-48045-2 (PMC11074293; doi:10.1038/s41467-024-48045-2)
Supplement: Supplementary file 2 — Description of Additional Supplementary Files [file 41467_2024_48045_MOESM2_ESM.pdf]

File Name: Supplementary Movie 1

Conformational dynamics of dimeric $CRL3^{KLHL22}$. The movie show that $CRL3^{KLHL22}$ transitioned from a compact conformation to a relaxed conformation.
