# Supplementary material for: A conserved N-terminal motif of CUL3 contributes to assembly and E3 ligase activity of CRL3KLHL22
Source: Nat Commun. 2024 May 6;15:3789. doi: 10.1038/s41467-024-48045-2 (PMC11074293; doi:10.1038/s41467-024-48045-2)
Supplement: Supplementary file 3 — Reporting Summary [file 41467_2024_48045_MOESM3_ESM.pdf]

nature portfolio

Corresponding author(s): Yuxin Yin

Last updated by author(s): Apr 6, 2024

Reporting Summary

Nature Portfolio wishes to improve the reproducibility of the work that we publish. This form provides structure for consistency and transparency in reporting. For further information on Nature Portfolio policies, see our Editorial Policies and the Editorial Policy Checklist.

Statistics

For all statistical analyses, confirm that the following items are present in the figure legend, table legend, main text, or Methods section.

n/a	Confirmed	
☐	☒	The exact sample size (*n*) for each experimental group/condition, given as a discrete number and unit of measurement
☒	☐	A statement on whether measurements were taken from distinct samples or whether the same sample was measured repeatedly
☒	☐	The statistical test(s) used AND whether they are one- or two-sided<br>*Only common tests should be described solely by name; describe more complex techniques in the Methods section.*
☒	☐	A description of all covariates tested
☒	☐	A description of any assumptions or corrections, such as tests of normality and adjustment for multiple comparisons
☐	☒	A full description of the statistical parameters including central tendency (e.g. means) or other basic estimates (e.g. regression coefficient) AND variation (e.g. standard deviation) or associated estimates of uncertainty (e.g. confidence intervals)
☒	☐	For null hypothesis testing, the test statistic (e.g. *F*, *t*, *r*) with confidence intervals, effect sizes, degrees of freedom and *P* value noted<br>*Give P values as exact values whenever suitable.*
☒	☐	For Bayesian analysis, information on the choice of priors and Markov chain Monte Carlo settings
☒	☐	For hierarchical and complex designs, identification of the appropriate level for tests and full reporting of outcomes
☒	☐	Estimates of effect sizes (e.g. Cohen's *d*, Pearson's *r*), indicating how they were calculated

Our web collection on statistics for biologists contains articles on many of the points above.

Software and code

Policy information about availability of computer code

Data collection	SerialEM 3.6.11; ASTRA(6.1.6.5); DiscoverMP (v2023 R2)
Data analysis	CryoSPARC(4.0.1), PHENIX(1.20.1), Coot(0.9.8.5), DeepEMhancer(0.14), Jalview(2.11.3.2), Clustal(2.0), UCSF Chimera(1.17.1), UCSF ChimeraX (1.7), Pymol(2.5.0), Gromacs(2023.2), VMD(1.9.4a57), Origin7.0,

For manuscripts utilizing custom algorithms or software that are central to the research but not yet described in published literature, software must be made available to editors and reviewers. We strongly encourage code deposition in a community repository (e.g. GitHub). See the Nature Portfolio guidelines for submitting code & software for further information.

Data

Policy information about availability of data

All manuscripts must include a data availability statement. This statement should provide the following information, where applicable:

- Accession codes, unique identifiers, or web links for publicly available datasets
- A description of any restrictions on data availability
- For clinical datasets or third party data, please ensure that the statement adheres to our policy

The cryo-EM density map of CUL3(WT)-RBX1-KLHL22 and CUL3(DEL1-24)-RBX1-KLHL22(1-178) have been deposited in the Electron Microscopy Data Bank under accession number EMD-36961 and EMD-36987, respectively. The corresponding atomic coordinates were deposited in the RCSB Protein Data Bank under accession numbers 8K8T and 8K9I. The crystal structure of CUL3-KLHL11, CUL3-KEAP1, CUL3-SPOP, and CUL3-A55 is obtained from the RCSB Protein Data Bank under accession numbers 4AP2, 5NLB, 4EOZ and 6I2M, respectively. The cryo-EM density map of CRL3-KLHL22 determined by Teng et al. (reference 37) was obtained from

Electron Microscopy Data Bank under accession number EMD-37247. Source data are provided with this paper.

Research involving human participants, their data, or biological material

Policy information about studies with human participants or human data. See also policy information about sex, gender (identity/presentation), and sexual orientation and race, ethnicity and racism.

Reporting on sex and gender	N/A
Reporting on race, ethnicity, or other socially relevant groupings	N/A
Population characteristics	N/A
Recruitment	N/A
Ethics oversight	N/A

Note that full information on the approval of the study protocol must also be provided in the manuscript.

Field-specific reporting

Please select the one below that is the best fit for your research. If you are not sure, read the appropriate sections before making your selection.

[x] Life sciences [ ] Behavioural & social sciences [ ] Ecological, evolutionary & environmental sciences

For a reference copy of the document with all sections, see nature.com/documents/nr-reporting-summary-flat.pdf

Life sciences study design

All studies must disclose on these points even when the disclosure is negative.

Sample size	Sufficient cryo-EM data were collected to achieve adequate map resolutions for model building. The number of independent experiments and biological replicates was indicated in each figure legend.
Data exclusions	Cryo-EM micrographs with ice or ethane contamination, empty carbon, and poor CTF fit (> 5 Å) were excluded manually. Particles belonging to bad classes were discarded and the data processing flowchart were summarized in Extended Data Fig. 2a and 6a. These criteria were preestablished and the procedure is a common practice in cryo-EM image analysis. No data was excluded in functional studies.
Replication	All attempts at replication were successful according to the detailed protocol described in the methods section. The numbers of replication were described in figure legends.
Randomization	For cryo-EM 3D refinement, all particles were randomly split into two groups. Randomization is not relevant to other experiments as no animal or human study was involved.
Blinding	The investigators were blinded to group allocation during cryo-EM data collection and analysis. Blinding is not relevant to other experiments as the investigator need to be aware of wild type CUL3 and CUL3 mutants.

Reporting for specific materials, systems and methods

We require information from authors about some types of materials, experimental systems and methods used in many studies. Here, indicate whether each material, system or method listed is relevant to your study. If you are not sure if a list item applies to your research, read the appropriate section before selecting a response.

Materials & experimental systems

n/a	Involved in the study	
[ ]	[x]	Antibodies
[ ]	[x]	Eukaryotic cell lines
[x]	[ ]	Palaeontology and archaeology
[x]	[ ]	Animals and other organisms
[x]	[ ]	Clinical data
[x]	[ ]	Dual use research of concern
[x]	[ ]	Plants

Methods

n/a	Involved in the study	
[x]	[ ]	ChIP-seq
[x]	[ ]	Flow cytometry
[x]	[ ]	MRI-based neuroimaging

Antibodies

Antibodies used	anti-CUL3 (Abcam, Ab75851, EPR3196Y, 1:1000), anti-CUL3 (Abclonal, A16455, 1:1000), anti-mCherry (Abcam, Ab213511, EPR20579, 1:1000), anti-KLHL22 (Proteintech, 16214-1-AP, 1:2000), anti-DEPDC5 (Abcam, Ab213181, EPR20497-23, 1:1000), anti-beta-actin (MBL, #PM053, 1:5000), anti-UBA1 (Abcam, ab181225, EPR14204(B), 1:5000), anti-KEAP1 (Abcam, Ab227828, EPR22664-26, 1:1000), anti-NEDD8 (Abcam, Ab81264, Y297, 1:1000), anti-Stag (Abcam, Ab180958, EPR12996, 1:1000), anti-UBE2D1 (Abclonal, A1951, 1:1000) or anti-MBP (Abcam, Ab119994, EPR4744, 1:5000), anti-Flag (Sigma, F3165, M2, 1:2000). All antibodies used are commercially available and extensively used.
Validation	Antibodies validation:<br>https://www.abcam.cn/products/primary-antibodies/cullin-3cul-3-antibody-epr3196y-ab75851.html<br>https://abclonal.com.cn/catalog/A16455<br>https://www.abcam.cn/products/primary-antibodies/mcherry-antibody-epr20579-ab213511.html<br>https://www.ptgcn.com/products/KLHL22-Antibody-16214-1-AP.htm<br>https://www.abcam.cn/products/primary-antibodies/depdc5-antibody-epr20497-23-ab213181.html<br>https://www.mblbio.com/bio/g/dtl/A/?pcd=PM053<br>https://www.abcam.cn/products/primary-antibodies/e1-ubiquitin-activating-enzyme-1uba1-antibody-epr14204b-ab181225.html<br>https://www.abcam.cn/products/primary-antibodies/keap1-antibody-epr22664-26-ab227828.html<br>https://www.abcam.cn/products/primary-antibodies/nedd8-antibody-y297-ab81264.html<br>https://www.abcam.cn/products/primary-antibodies/s-tag-antibody-epr12996-ab180958.html<br>https://abclonal.com.cn/catalog/A1951<br>https://www.abcam.cn/products/primary-antibodies/maltose-binding-protein-antibody-epr4744-ab119994.html<br>https://www.sigmaaldrich.cn/CN/en/product/sigma/f3165

Eukaryotic cell lines

Policy information about cell lines and Sex and Gender in Research

Cell line source(s)	Sf9 and HEK 293T cells were from Thermo Fisher Scientific.
Authentication	None of the cell line used was authenticated.
Mycoplasma contamination	All cell lines were tested negative for mycoplasma contamination.
Commonly misidentified lines (See ICLAC register)	No commonly misidentified cell lines were used.

Plants

Seed stocks	*Report on the source of all seed stocks or other plant material used. If applicable, state the seed stock centre and catalogue number. If plant specimens were collected from the field, describe the collection location, date and sampling procedures.*
Novel plant genotypes	*Describe the methods by which all novel plant genotypes were produced. This includes those generated by transgenic approaches, gene editing, chemical/radiation-based mutagenesis and hybridization. For transgenic lines, describe the transformation method, the number of independent lines analyzed and the generation upon which experiments were performed. For gene-edited lines, describe the editor used, the endogenous sequence targeted for editing, the targeting guide RNA sequence (if applicable) and how the editor was applied.*
Authentication	*Describe any authentication procedures for each seed stock used or novel genotype generated. Describe any experiments used to assess the effect of a mutation and, where applicable, how potential secondary effects (e.g. second site T-DNA insertions, mosiacism, off-target gene editing) were examined.*
